# Parkour-Based Activities in the Athletic Development of Youth Basketball Players

**DOI:** 10.3389/fphys.2021.771368

**Published:** 2021-10-15

**Authors:** Mark David Williams, Ben William Strafford, Joseph Antony Stone, Jason Moran

**Affiliations:** ^1^School of Sport, Rehabilitation and Exercise Sciences, University of Essex, Colchester, United Kingdom; ^2^Sport and Physical Activity Research Centre, Sheffield Hallam University, Sheffield, United Kingdom

**Keywords:** fundamental movement skills, non-linear pedagogy, youth athletes, strength training, affordance landscape

## Abstract

While ideas from long-term athlete development (LTAD) models have been adopted and integrated across different sports, issues related to early specialization, such as increased risk of injury and burnout, are still common. Although some benefits may be associated with early sport specialization, sports sampling is purported to be a more effective approach to the long-term health and wellbeing of children. Furthermore, the concept of developing what are commonly referred to as “fundamental movement skills” (FMS) is central to the rationale for delaying single sports specialization. However, in place of sports sampling, it appears that the practice of strength and conditioning (S&C) has become a driving force behind developmental models for youth athletes, highlighted by the growing body of literature regarding youth athletic development training. In this perspective piece, we explore how conventional S&C practice may insufficiently develop FMS because typically, it only emphasizes a narrow range of foundational exercises that serve a limited role toward the development of action capabilities in youth athletic populations. We further discuss how this approach may limit the transferability of physical qualities, such as muscular strength, to sports-specific tasks. Through an ecological dynamics lens, and using basketball as an example, we explore the potential for parkour-based activity within the LTAD of youth basketball players. We propose parkour as a training modality to not only encourage movement diversity and adaptability, but also as part of an advanced strength training strategy for the transfer of conventional S&C training.

## Introduction

The notion of developing basic movement skills to provide foundations for more advanced and specialized forms of movement is not new (Hulteen et al., 2018). However, a concern in the development of youth sports has been the lack of emphasis on generalized fundamental movement skills (FMS) in favor of early specialization (Bridge and Toms, 2013; DiStefano et al., 2017; Liefeith et al., 2018). Although alternative terms exist (e.g., foundational movement skills, functional movement skills, and basic movement skills), typically, FMS encompass locomotor (e.g., running and jumping) and object control (e.g., catching, throwing, and kicking; Morgan et al., 2013; Barnett et al., 2016). Accordingly, FMS are considered foundational for the development of sports-specific skills, which if left undeveloped may limit future performance (Barela, 2013; Arede et al., 2019; Jukic et al., 2019). Indeed, the development of FMS ahead of specific sports skills is promoted within the long-term athlete development model (LTAD; Balyi, 2001), which has served as an influential framework for the training of young athletes in sporting organizations for over two decades (Collins and Bailey, 2012; Liefeith et al., 2018; Perreault and Gonzalez, 2021).

Through the development of FMS as well as participation in multiple sports-related activities throughout childhood, the premise of the LTAD model is to avoid early specialization and the associated risks relating to injury and burnout (Ford et al., 2011; Pichardo et al., 2018; Perreault and Gonzalez, 2021). However, despite recognition by sports organizations of the need for an LTAD strategy, the prevalence of injuries in youth sports, such as soccer and basketball, remains high (e.g., Read et al., 2016, 2018; Owoeye et al., 2020). While the original intention of the LTAD model was to be used as a framework for sports organizations to adapt and implement to suit their specific needs (Dowling et al., 2020), it has been argued that the development of FMS and general physical qualities remains marginalized in favor of sports-specific training (Liefeith et al., 2018; Williams et al., 2021).

Problematically, much debate exits with respect to FMS (e.g., Barnett et al., 2016; Hulteen et al., 2018; Newell, 2020). Indeed, youth-level basketball coaches have been found to have differing notions of FMS, as well as varying ideas as to whom might be responsible for their development (Williams et al., 2021). Consequently, sports organizations may have become reliant on the field of strength and conditioning (S&C) to develop FMS and general physical qualities. For example, within Basketball England’s version of the LTAD model, the *Player Development Framework*, the S&C domain is responsible for the development of “all round quality of movement literacy.” In relation to this, the meta-analysis by Collins et al. (2019) found that resistance training, which targets muscular strength, positively impacts FMS through neural adaptations (e.g., motor unit recruitment and firing). However, despite the benefits of youth-based S&C training, which includes reducing risk factors for injury and life-long engagement in physical activity (e.g., Faigenbaum et al., 2013; Zwolski et al., 2017; McQuilliam et al., 2020), conventional youth-based S&C practices may lead to the development of movement skills with limited relevance outside of the S&C domain. For example, the development of *athletic movement skills*, such as the overhead squat, hip hinge, and lunge patterns (Woods et al., 2017). Consequently, FMS may not be developed with sufficient diversity to provide underpinning movement capabilities for sports-specific skill development (Young, 2006; Young et al., 2015).

A potential strategy to enrich young athletes’ FMS education is the implementation of parkour-related activities (Strafford et al., 2018, 2020). Parkour is an acrobatic sport incorporating a broad range of movement skills and motor abilities, which has been proposed as an activity to develop FMS and general athletic abilities for youth team sports (Strafford et al., 2018, 2020; Wormhoudt et al., 2018). Obtaining transferable athletic capabilities through the implementation of parkour derives from the concept of *donor sports*, which are purported to develop and facilitate the transfer of general movement skills and physical qualities to actions typically performed in a *target sport* (Travassos et al., 2018; Wormhoudt et al., 2018). Given that basketball is characterized by multidirectional movements (Montgomery et al., 2010), the development of youth basketball players would seemingly benefit from the running, jumping, vaulting, and climbing activities that characterize parkour (DeMartini, 2014).

Thus, in this perspective article, we explore the potential for parkour as a donor sport for the development of youth basketball players. In the next sections, we discuss the role of conventional youth-based S&C practice and its limitations, and present alternative perspectives on the development of movement capabilities through an ecological dynamics lens. It is through this lens that we propose parkour as a donor sport for the enriched development of FMS, as well as forming an advanced strength training strategy to facilitate transfer to basketball performance.

## The Role of Strength and Conditioning in LTAD

A body of research (e.g., DiStefano et al., 2010; Myer et al., 2011; Ayala et al., 2017; Pomares-Noguera et al., 2018) has demonstrated the efficacy of neuromuscular training programs on reducing risk factors for injury in youth populations. Furthermore, other forms of S&C training in youth populations are also supported empirically (Moran et al., 2018a,b, 2019). This includes evidence of windows of trainability for strength, speed, and plyometrics (Moran et al., 2018a,b, 2019). Collectively, this has resulted in the publication of position papers, such as the *National Strength and Conditioning Association’s* LTAD position statement and the *British Journal of Sports Medicine’s* position statement on youth resistance training, both of which recommend the concurrent development of muscular strength and movement skills in children and adolescents (Lloyd et al., 2014, 2016). Therefore, the role of S&C within the LTAD strategies of sports organizations should be regarded as highly important in reducing risk factors for injury as well as increasing physical performance capabilities (Faigenbaum et al., 2013; Zwolski et al., 2017; Pichardo et al., 2018).

Notwithstanding the aforementioned benefits, a concern relating to the conventional approach to youth-based S&C is the lack of representative movement dynamics for team sports, such as basketball. Indeed, when considered in the context of “open-skill” games that require decision making and a vast array of movement dynamics (Smith, 2016), athletic movement skills may not sufficiently reflect the requirements. To illustrate this, in basketball, offensive players require a large repertoire of action capabilities to evade their opponents, as do defending players who are required to react (Montgomery et al., 2010). Accordingly, it has been argued that to be effective, S&C programs for basketball players need to better represent the diversity of movement demands of the sport (Taylor et al., 2015). This contention may also include plyometric exercise, which provides a stimulus to improve jumping, sprinting, and change of direction capabilities through enhancement of the stretch-shortening cycle (Hernández et al., 2018; Ramirez-Campillo et al., 2020). Although these physical qualities are specific to basketball (Ramirez-Campillo et al., 2020), it has been argued that the importance of the strength-related qualities of agility performance is relatively diminished against the perceptual and decision-making components (Young et al., 2015). Moreover, youth guidelines relating to the prescription of plyometric exercise appear to limit the scope for movement diversity by placing an emphasis on technical proficiency in exercises, such as “in-place hops” ahead of progression to more elaborate jumping variations (Cronin and Radnor, 2019). While the safety of young athletes is of paramount importance, the youth guidelines for plyometric training may serve to discourage exploration and development of jumping skills that are more characteristic of sports, such as basketball.

Without devaluing the importance of conventional S&C training, it may be that despite its emphasis on developing broad FMS within the LTAD framework, there is scope to encourage a vaster array of action capabilities. We propose that the S&C domain further permeates the development of youth athletes by more thoroughly accounting for the decision-making properties and diverse array of movement dynamics that characterizes skilled motor performance. Accordingly, we consider the merit in adopting an ecological dynamics approach to motor learning.

## Adopting an Ecological Dynamics Perspective

The ecological dynamics framework is formed from both ecological psychology and dynamics systems theory (O’Sullivan et al., 2020; Rudd et al., 2020). Through the ecological psychology lens, information perceived from the environment specifies the parameters that dictate how a skill is performed (Frère and Hug, 2012). The opportunities for action that an individual perceives from their environment represent what is termed the *affordance landscape* (Davids, 2012; Heras-Escribano and De Pinedo-García, 2018; Savelsbergh and Wormhoudt, 2018). For example, a basketball player preparing to shoot will perceive information relating to the proximity of the defensive player, their own location on the court, and the time left on the shot clock. Collectively, this information will influence the dynamics of the shot with respect to the kinetics and kinematics (Gorman and Maloney, 2016). In a second example, a player in possession of the ball may detect the space between defenders as an opportunity to dribble and *drive* through to advance toward the basket. In this example, based upon the defenders positioning, the attacking player has different action possibilities (affordances) in regard to the direction they may drive (Esteves et al., 2011). Thus, perception of the environment and the subsequent action are considered to be coupled (Smith, 2016).

Within ecological dynamics, in place of fixed movement patterns, the ever-changing nature of information from the environment requires adaptability from the performer to coordinate the appropriate action (Davids et al., 2013; Rudd et al., 2020). In contrast to fixed movement patterns, muscle synergies, which represent neural organizations, enable a vast array of adaptable movement possibilities (Frère and Hug, 2012; Latash, 2012; Bizzi and Cheung, 2013). This is particularly pertinent to how adjustments to an ongoing movement skill occur in response to perturbations (e.g., unexpected changes to surfaces; Newell, 1991; Smith, 2016). Contributing to the vast array of action capabilities is the combination of anatomical characteristics, learned coordinative patterns, and changes to physical output (e.g., force production and stretch-shortening properties), which form an individual’s *effectivities* (Witt and Riley, 2014; Wang and Bingham, 2019). Importantly, properties that form effectivities are continually altered across developmental stages of growth and maturation (Ribeiro et al., 2021), in turn necessitating the continual exploration of the affordance landscape with respect to an individual’s action capabilities.

## The Potential of Parkour

Despite popular media portraying parkour as an extreme sport consisting of only large-scale movements that are of high injury risk, such as jumping from buildings or between train carriages (Strafford et al., 2018), expert *Traceurs* have highlighted how contemporary parkour consists of a range of events (e.g., speed-runs and freestyle) which can be performed both in indoor and outdoor environments (Strafford et al., 2020). Hence, parkour is characterized by a variety of movements utilized to navigate obstacles and is practiced in various forms and contexts (Aggerholm and Højbjerre Larsen, 2017). The potential of parkour to enrich FMS is based upon the concept of donor sports, which is derived from the Athletic Skills Model (ASM; Wormhoudt et al., 2018). The ASM, which adopts an ecological dynamics perspective, purports that exposure to activities that share common characteristics (e.g., skills and abilities) can be transferred or “donated” to a target sport (Strafford et al., 2018; Rudd et al., 2020). Parkour invites different ways of moving based upon the performer’s perception of surroundings and promotes creativity to navigate gaps and obstacles (Aggerholm and Højbjerre Larsen, 2017; Rudd et al., 2020). Given these characteristics, Strafford et al. (2018) propose that the incorporation of parkour-related activities could provide a platform for youth athletes to develop FMS that could be transferred to other sports. For example, the use of obstacle courses, termed *speed-runs*, which require the participant to navigate as efficiently as possible, can be used to encourage transferable agility skills (Strafford et al., 2021). Indeed, irrespective of the target sport, exposure to parkour-based activities, such as speed-runs, may be particularly pertinent during pre-adolescence, which is regarded as a period of sensitivity for developing FMS due to high levels of neural plasticity (Myer et al., 2015; Ng and Button, 2018). However, for the purposes of *fine tune* existing neural pathways and muscle synergies, and to take advantage of the still high levels of neural plasticity retained in adolescence (~13years of age and above; Myer et al., 2013, 2015), parkour-based activities may continue to play an important role in athletic development.

Although currently, evidence directly examining the benefits of parkour training on basketball is limited, significant correlations between performance tests typically used in basketball (e.g., vertical jump and T-test) and performance in a parkour speed-run has been demonstrated (Strafford et al., 2021). Furthermore, Abellán-Aynés and Alacid (2016) present parkour as an effective training method for developing agility, horizontal, and vertical jump abilities. Alongside jumping and agility, parkour training interventions have also demonstrated improved cardiorespiratory fitness with increases in peak oxygen uptake, oxygen uptake at anaerobic threshold, heart rate at anaerobic threshold, and running speed at anaerobic threshold (Dvorak et al., 2017).

Regarding basketball, owing to similarities between actions, parkour-based activities may also be considered for their potential as a donor for the specific development of action capabilities in youth players. For example, in parkour, the *tic tac* action, which is characterized by pushing off of a wall with the ball of the foot to gain height (Witfeld et al., 2011), requires spatial orientation and use of perceptual information from the foot contact to determine the subsequent phase of the movement (Strafford et al., 2018). Therefore, this action may present developing basketball players with the opportunity to explore their capabilities to decelerate, propel, land, and then, move in a new direction. Furthermore, through what has been termed “synergistic adaptation,” the introduction of strength training to youth basketball players will likely augment changes to force production that naturally occur as a result of growth and maturation (Moran et al., 2017; Peitz et al., 2018). In turn, this will alter the players’ effectivities (force capabilities), which necessitates the continued exploration of the affordance landscape with respect to their action capabilities. To illustrate this, the use of plyometric training, which has been found to enhance the jumping capabilities of youth basketball players (Gonzalo-Skok et al., 2019), logically, enables players to express improved jumping capabilities within the game. For example, in the execution of rebounding the ball. Rebounding involves an offensive or defensive player aerially competing for possession of the ball after a missed shot attempt. However, depending upon the specific scenario presented, the player may be required to use various jumping actions to successfully rebound the ball (Krause and Nelson, 2018). Therefore, despite a player’s enhanced force characteristics, in the absence of the players exploring their jump action capabilities beyond the plyometric regimen, there may be a limited transfer of the adaptations to sport-specific contexts. In this regard, parkour-based actions need not be advanced beyond those identified as relevant to the affordance landscape. Instead, the actions remain efficacious for the process of *recalibration*, which represents an updating of the mapping of the contributing units to the execution of a movement skill (Davids et al., 2012).

Although it may be argued that basketball-specific practice would better facilitate transfer of improved force-related capabilities, problematically, the greater levels of representativeness that basketball-specific practice presents may provide cognitive and decision-making demands that are too high (Farrow and Robertson, 2017). Therefore, youth players may fail to sufficiently explore the affordance landscape in relation their altered physical capabilities. This is not to appear contradictory to the premises of ecological dynamics already considered in regard to the coupling of perception and action; instead, it distinguishes between the effectivities (those impacted by S&C) of the individual player and the more complex environment that represents the sport (Woods et al., 2020). In this regard, affordances are both objective, for example, the properties of a given playing surface, and subjective, which relate to an individual’s perception of their own capabilities (Davids et al., 2008). With reference to the latter, the detection of affordances therefore relates to an athlete’s current effectivities (Wang and Bingham, 2019; Ribeiro et al., 2021). Where the properties of effectivities are enhanced through conventional S&C training, parkour movement training is proposed to sit between conventional S&C training and that of basketball-specific training. However, as with any training modality, caution should be exercised to avoid excessive workload being placed upon youth athletes, especially in the form of repetitive movement patterns (Leppänen et al., 2015). Notwithstanding this, when programmed appropriately, theoretically, the inclusion of parkour-based activities would enable the youth player to perceive their action capabilities and detect new affordances transferable to their sport.

## Application as an Advanced Strength Training Strategy

An important consideration in the development of adolescent basketball players is that the number of basketball-specific practice hours will generally increase proportional to the time spent in other physical activities (Jayanthi et al., 2013). Therefore, the inclusion of parkour activities will likely be dependent on the constraints of time. Accordingly, at this stage of development, the use of parkour activities might form part of a more advanced strength training strategy and adopt a more thoughtful and individually tailored approach. In this regard, parkour activities should be considered by S&C coaches alongside an evaluation of the specific sporting action being targeted.

To account for time constraints, parkour activities could theoretically be embedded within the S&C program itself. For example, this could take the form of a complex training regimen, with parkour actions performed concurrently within the same training session as conventional S&C training exercises. Complex training has previously been shown as an effective method to improve sprint and vertical jump performance in young (<20years) basketball players (Santos and Janeira, 2008; Freitas et al., 2017). Commonly, this training method requires athletes to perform a strength-oriented exercise, such as a barbell back squat followed by a plyometric-oriented exercise that shares similar mechanics, therefore providing a potentiating effect on the subsequent exercise (Santos and Janeira, 2008). Where the paired exercise in this example would typically include a jumping exercise, such as a countermovement jump (Freitas et al., 2017), vaulting activities or tic tac actions could be included in its place, or in combination through alternating sets. With regard to the latter, from an ecological dynamics perspective, this approach would challenge players to explore the affordance landscape under conditions of the post-activation potentiation response from the strength-oriented exercise, augmenting the neural contribution to the subsequent parkour action in each set of the exercise, as is the aim of complex training (Freitas et al., 2017). Moreover, the varied jumping patterns would present players with more varied landing challenges than those in conventional complex training, which may better prepare players for scenarios encountered within the sport. While currently, no known loading parameters exit for parkour-based actions, it would appear prudent to follow the guidelines for contacts that are typical of plyometric and complex training regimens. However, research is required to validate these suppositions.

## Safety Precautions

*Parkour UK*, the governing body for parkour in the United Kingdom, has developed its own risk-benefit assessment and provides standards relating to equipment and codes of practice. However, its growing popularity is illustrated by the emergence of YouTube videos displaying high-risk maneuvers in urban settings (DeMartini, 2014). Therefore, where parkour actions are being considered within the LTAD programs of young athletes, risk-benefit should be considered, and an emphasis placed on performing parkour safely. Moreover, when introduced, it should be stressed to the young athletes that the parkour activities are to be performed in supervised sessions only.

## Concluding Remarks

Given the S&C domain’s influence in the LTAD of youth athletic populations, we propose that the field expands its influence to capture both the decision-making and movement dynamics properties that may better represent the characteristics of sports performance. While the efficacy of conventional S&C is not in question, we have argued that through the adoption of concepts from the ecological dynamics’ framework, the S&C domain might better equip children and adolescents with diverse and adaptable action capabilities. Moreover, this would develop perceptual aspects of performance, and the interdependency of environment and movement dynamics. From this perspective, the implementation of parkour as a donor sport for youth basketball players might enrich their action capabilities and facilitate the transfer of conventional forms of S&C to basketball performance.

## Data Availability Statement

The original contributions presented in the study are included in the article/supplementary material, and further inquiries can be directed to the corresponding author.

## Author Contributions

All authors contributed to the writing of the manuscript. MW and BS conceived the presented idea. MW developed the application of parkour to basketball and how strength training modalities could be advanced with parkour-based activities. MW took the lead on writing the manuscript with BS very much influencing the work and contributing to the writing of the parkour and ecological dynamics aspects of the manuscript. JM provided critical thought relating to the LTAD and strength training elements. JM also contributed to the critical analysis and writing of the manuscript. JS contributed to the shape of the work and provided input relating to the ecological dynamics framework.

## Conflict of Interest

The authors declare that the research was conducted in the absence of any commercial or financial relationships that could be construed as a potential conflict of interest.

The reviewer DB declared a past co-authorship with one of the authors JM.

## Publisher’s Note

All claims expressed in this article are solely those of the authors and do not necessarily represent those of their affiliated organizations, or those of the publisher, the editors and the reviewers. Any product that may be evaluated in this article, or claim that may be made by its manufacturer, is not guaranteed or endorsed by the publisher.

## References

[ref1] Abellán-Aynés O. Alacid F. (2016). Anthropometric profile, physical fitness and differences between performance level of parkour practitioners. Arch. Med. Deporte 33, 312–316.

[ref2] Aggerholm K. Højbjerre Larsen S. (2017). Parkour as acrobatics: an existential phenomenological study of movement in parkour. Qual. Res. Sport Exerc. Health 9, 69–86. doi: 10.1080/2159676X.2016.1196387

[ref3] Arede J. Esteves P. Ferreira A. P. Sampaio J. Leite N. (2019). Jump higher, run faster: effects of diversified sport participation on talent identification and selection in youth basketball. J. Sports Sci. 37, 2220–2227. doi: 10.1080/02640414.2019.1626114, PMID: 31164046

[ref4] Ayala F. Pomares-Noguera C. Robles-Palazón F. del Pilar García-Vaquero M. Ruiz-Pérez I. Hernández-Sánchez S.. (2017). Training effects of the FIFA 11+ and Harmoknee on several neuromuscular parameters of physical performance measures. Int. J. Sports Med. 38, 278–289. doi: 10.1055/s-0042-121260, PMID: 28192831

[ref5] Balyi I. (2001). Sport system building and long-term athlete development in British Columbia. Coach. Rep. 8, 22–28.

[ref6] Barela J. A. (2013). Fundamental motor skill proficiency is necessary for children’s motor activity inclusion. Motriz Rev. Educ. Fis. 19, 548–551. doi: 10.1590/S1980-65742013000300003

[ref7] Barnett L. M. Stodden D. Cohen K. E. Smith J. J. Lubans D. R. Lenoir M.. (2016). Fundamental movement skills: an important focus. J. Teach. Phys. Educ. 35, 219–225. doi: 10.1123/jtpe.2014-0209

[ref8] Bizzi E. Cheung V. C. (2013). The neural origin of muscle synergies. Front. Comput. Neurosci. 7:51. doi: 10.3389/fncom.2013.00051, PMID: 23641212 PMC3638124

[ref9] Bridge M. W. Toms M. R. (2013). The specialising or sampling debate: a retrospective analysis of adolescent sports participation in the UK. J. Sports Sci. 31, 87–96. doi: 10.1080/02640414.2012.721560, PMID: 22974248

[ref10] Collins D. Bailey R. (2012). ‘Scienciness’ and the allure of second-hand strategy in talent identification and development. Int. J. Sport Policy 5, 1–9. doi: 10.1080/19406940.2012.656682

[ref11] Collins H. Booth J. N. Duncan A. Fawkner S. (2019). The effect of resistance training interventions on fundamental movement skills in youth: a meta-analysis. Sports Med. Open 5:17. doi: 10.1186/s40798-019-0188-x, PMID: 31102027 PMC6525228

[ref12] Cronin J. B. Radnor J. M. (2019). “Plyometric training for youth athletes,” in Strength and Conditioning for Youth Athletes. 2nd Edn. eds. Lloyd R. S. Oliver J. L. (Oxfordshire, England: Routledge), 188–207.

[ref13] Davids K. (2012). Principles of motor learning in ecological dynamics: a comment on functions of learning and the acquisition of motor skills (with reference to sport). Open Sports Sci. J. 5, 113–117. doi: 10.2174/1875399X01205010113

[ref14] Davids K. Araújo D. Hristovski R. Chow J. Y. (2012). “Ecological dynamics and motor learning design in sport,” in Skill Acquisition in Sport: Research, Theory & Practice. 2nd Edn. eds. Williams M. Hodges N.. (Oxfordshire, England: Routledge), 112–130.

[ref15] Davids K. Araújo D. Vilar L. Renshaw I. Pinder R. (2013). An ecological dynamics approach to skill acquisition: implications for development of talent in sport. Talent Dev. Excell. 5, 21–34.

[ref16] Davids K. Button C. Bennett S. (2008). Dynamics of Skill Acquisition: A Constraints-Led Approach. Champaign, Illinois: Human Kinetics.

[ref17] DeMartini A. L. (2014). Is parkour a problem? College and university liability for extreme sport activities. Recreational Sports J. 38, 69–81. doi: 10.1123/rsj.2014-0039

[ref18] DiStefano L. J. Beltz E. M. Root H. J. Martinez J. C. Houghton A. Taranto N.. (2017). Sport sampling is associated with improved landing technique in youth athletes. Sports Health 10, 160–168. doi: 10.1177/1941738117736056, PMID: 29131779 PMC5857728

[ref19] DiStefano L. J. Padua D. A. Blackburn J. T. Garrett W. E. Guskiewicz K. M. Marshall S. W. (2010). Integrated injury prevention program improves balance and vertical jump height in children. J. Strength Cond. Res. 24, 332–342. doi: 10.1519/JSC.0b013e3181cc2225, PMID: 20072067

[ref20] Dowling M. Mills J. Stodter A. (2020). Problematizing the adoption and implementation of athlete development ‘models’: a Foucauldian-inspired analysis of the long-term athlete development framework. J. Athl. Dev. Exp. 2:3. doi: 10.25035/jade.02.03.03

[ref21] Dvorak M. Eves N. Bunc V. Balas J. (2017). Effects of parkour training on health-related physical fitness in male adolescents. Open Sports Sci. J. 10, 132–140. doi: 10.2174/1875399X01710010132

[ref22] Esteves P. T. de Oliveira R. F. Araújo D. (2011). Posture-related affordances guide attacks in basketball. Psychol. Sport Exerc. 12, 639–644. doi: 10.1016/j.psychsport.2011.06.007

[ref23] Faigenbaum A. D. Lloyd R. S. Myer G. D. (2013). Youth resistance training: past practices, new perspectives, and future directions. Pediatr. Exerc. Sci. 25, 591–604. doi: 10.1123/pes.25.4.591, PMID: 24214441

[ref24] Farrow D. Robertson S. (2017). Development of a skill acquisition periodisation framework for high-performance sport. Sports Med. 47, 1043–1054. doi: 10.1007/s40279-016-0646-2, PMID: 27873190

[ref25] Ford P. De Ste Croix M. Lloyd R. Meyers R. Moosavi M. Oliver J.. (2011). The long-term athlete development model: physiological evidence and application. J. Sports Sci. 29, 389–402. doi: 10.1080/02640414.2010.536849, PMID: 21259156

[ref26] Freitas T. T. Martinez-Rodriguez A. Calleja-González J. Alcaraz P. E. (2017). Short-term adaptations following complex training in team-sports: A meta-analysis. PLoS One 12:e0180223. doi: 10.1371/journal.pone.0180223, PMID: 28662108 PMC5491153

[ref27] Frère J. Hug F. (2012). Between-subject variability of muscle synergies during a complex motor skill. Front. Comput. Neurosci. 6:99. doi: 10.3389/fncom.2012.00099, PMID: 23293599 PMC3531715

[ref28] Gonzalo-Skok O. Sánchez-Sabaté J. Izquierdo-Lupón L. Sáez de Villarreal E. (2019). Influence of force-vector and force application plyometric training in young elite basketball players. Eur. J. Sport Sci. 19, 305–314. doi: 10.1080/17461391.2018.1502357, PMID: 30058461

[ref29] Gorman A. D. Maloney M. A. (2016). Representative design: does the addition of a defender change the execution of a basketball shot? Psychol. Sport Exerc. 27, 112–119. doi: 10.1016/j.psychsport.2016.08.003

[ref30] Heras-Escribano M. De Pinedo-García M. (2018). Affordances and landscapes: overcoming the nature–culture dichotomy through niche construction theory. Front. Psychol. 8:2294. doi: 10.3389/fpsyg.2017.02294, PMID: 29375426 PMC5767241

[ref31] Hernández S. Ramirez-Campillo R. Álvarez C. Sanchez-Sanchez J. Moran J. Pereira L. A.. (2018). Effects of plyometric training on neuromuscular performance in youth basketball players: a pilot study on the influence of drill randomization. J. Sports Sci. Med. 17, 372–378. PMID: 30116110 PMC6090388

[ref32] Hulteen R. M. Morgan P. J. Barnett L. M. Stodden D. F. Lubans D. R. (2018). Development of foundational movement skills: a conceptual model for physical activity across the lifespan. Sports Med. 48, 1533–1540. doi: 10.1007/s40279-018-0892-6, PMID: 29524160

[ref33] Jayanthi N. Pinkham C. Dugas L. Patrick B. LaBella C. (2013). Sports specialization in young athletes. Sports Health 5, 251–257. doi: 10.1177/1941738112464626, PMID: 24427397 PMC3658407

[ref34] Jukic I. Prnjak K. Zoellner A. Tufano J. J. Sekulic D. Salaj S. (2019). The importance of fundamental motor skills in identifying differences in performance levels of U10 soccer players. Sports 7:178. doi: 10.3390/sports7070178, PMID: 31336618 PMC6680693

[ref35] Krause J. Nelson C. (2018). Basketball Skills & Drills. 4th Edn. Champaign, Illinois: Human Kinetics.

[ref36] Latash M. L. (2012). The bliss (not the problem) of motor abundance (not redundancy). Exp. Brain Res. 217, 1–5. doi: 10.1007/s00221-012-3000-4, PMID: 22246105 PMC3532046

[ref37] Leppänen M. Pasanen K. Kujala U. M. Parkkari J. (2015). Overuse injuries in youth basketball and floorball. Open Access J. Sports Med. 6, 173–179. doi: 10.2147/OAJSM.S82305, PMID: 26045679 PMC4447174

[ref38] Liefeith A. Kiely J. Collins D. Richards J. (2018). Back to the future – in support of a renewed emphasis on generic agility training within sports-specific developmental pathways. J. Sports Sci. 36, 2250–2255. doi: 10.1080/02640414.2018.1449088, PMID: 29521175

[ref39] Lloyd R. S. Cronin J. B. Faigenbaum A. D. Haff G. G. Howard R. Kraemer W. J.. (2016). National strength and conditioning association position statement on long-term athletic development. J. Strength Cond. Res. 30, 1491–1509. doi: 10.1519/JSC.0000000000001387, PMID: 26933920

[ref40] Lloyd R. S. Faigenbaum A. D. Stone M. H. Oliver J. L. Jeffreys I. Moody J. A.. (2014). Position statement on youth resistance training: the 2014 international consensus. Br. J. Sports Med. 48, 498–505. doi: 10.1136/bjsports-2013-092952, PMID: 24055781

[ref41] McQuilliam S. J. Clark D. R. Erskine R. M. Brownlee T. E. (2020). Free-weight resistance training in youth athletes: a narrative review. Sports Med. 50, 1567–1580. doi: 10.1007/s40279-020-01307-7, PMID: 32578028 PMC7441088

[ref42] Montgomery P. G. Pyne D. B. Minahan C. L. (2010). The physical and physiological demands of basketball training and competition. Int. J. Sports Physiol. Perform. 5, 75–86. doi: 10.1123/ijspp.5.1.75, PMID: 20308698

[ref43] Moran J. Clark C. C. T. Ramirez-Campillo R. Davies M. J. Drury B. (2019). A meta-analysis of plyometric training in female youth: its efficacy and shortcomings in the literature. J. Strength Cond. Res. 33, 1996–2008. doi: 10.1519/JSC.0000000000002768, PMID: 30052601

[ref44] Moran J. Parry D. A. Lewis I. Collison J. Rumpf M. C. Sandercock G. R. H. (2018a). Maturation-related adaptations in running speed in response to sprint training in youth soccer players. J. Sci. Med. Sport 21, 538–542. doi: 10.1016/j.jsams.2017.09.012, PMID: 28964690

[ref45] Moran J. Sandercock G. Ramirez-Campillo R. Clark C. C. T. Fernandes J. F. T. Drury B. (2018b). A meta-analysis of resistance training in female youth: its effect on muscular strength, and shortcomings in the literature. Sports Med. 48, 1661–1671. doi: 10.1007/s40279-018-0914-4, PMID: 29626334

[ref46] Moran J. Sandercock G. R. H. Ramírez-Campillo R. Meylan C. Collison J. Parry D. A. (2017). A meta-analysis of maturation-related variation in adolescent boy athletes’ adaptations to short-term resistance training. J. Sports Sci. 35, 1041–1051. doi: 10.1080/02640414.2016.1209306, PMID: 27454545

[ref47] Morgan P. J. Barnett L. M. Cliff D. P. Okely A. D. Scott H. A. Cohen K. E.. (2013). Fundamental movement skill interventions in youth: a systematic review and meta-analysis. Pediatrics 132, e1361–e1383. doi: 10.1542/peds.2013-1167, PMID: 24167179

[ref48] Myer G. D. Faigenbaum A. D. Edwards N. M. Clark J. F. Best T. M. Sallis R. E. (2015). Sixty minutes of what? A developing brain perspective for activating children with an integrative exercise approach. Br. J. Sports Med. 49, 1510–1516. doi: 10.1136/bjsports-2014-093661, PMID: 25617423

[ref49] Myer G. D. Faigenbaum A. D. Ford K. R. Best T. M. Bergeron M. F. Hewett T. E. (2011). When to initiate integrative neuromuscular training to reduce sports-related injuries in youth? Curr. Sports Med. Rep. 10, 155–166. doi: 10.1249/JSR.0b013e31821b1442, PMID: 21623307 PMC3105332

[ref50] Myer G. D. Kushner A. M. Faigenbaum A. D. Kiefer A. Kashikar-Zuck S. Clark J. F. (2013). Training the developing brain, part I: cognitive developmental considerations for training youth. Curr. Sports Med. Rep. 12, 304–310. doi: 10.1097/01.CSMR.0000434106.12813.69, PMID: 24030303

[ref51] Newell K. M. (1991). Motor skill acquisition. Annu. Rev. Psychol. 42, 213–237. doi: 10.1146/annurev.ps.42.020191.001241, PMID: 2018394

[ref52] Newell K. M. (2020). What are fundamental motor skills and what is fundamental about them? J. Mot. Learn. Dev. 8, 280–314. doi: 10.1123/jmld.2020-0013

[ref53] Ng J. L. Button C. (2018). Reconsidering the fundamental movement skills construct: implications for assessment. Mov. Sport Sci. Sci. Mot. 102, 19–29. doi: 10.1051/sm/2018025

[ref54] O’Sullivan M. Davids K. Woods C. T. Rothwell M. Rudd J. (2020). Conceptualizing physical literacy within an ecological dynamics framework. Quest 72, 448–462. doi: 10.1080/00336297.2020.1799828

[ref55] Owoeye O. B. A. Ghali B. Befus K. Stilling C. Hogg A. Choi J.. (2020). Epidemiology of all-complaint injuries in youth basketball. Scand. J. Med. Sci. Sports 30, 2466–2476. doi: 10.1111/sms.13813, PMID: 32846028

[ref56] Peitz M. Behringer M. Granacher U. (2018). A systematic review on the effects of resistance and plyometric training on physical fitness in youth - what do comparative studies tell us? PLoS One 13:e0205525. doi: 10.1371/journal.pone.0205525, PMID: 30304033 PMC6179270

[ref57] Perreault M. E. Gonzalez S. P. (2021). Generalize over specialize: examining the long-term athlete development model to optimize youth athlete development. Strategies 34, 11–15. doi: 10.1080/08924562.2021.1896914

[ref58] Pichardo A. W. Oliver J. L. Harrison C. B. Maulder P. S. Lloyd R. S. (2018). Integrating models of long-term athletic development to maximize the physical development of youth. Int. J. Sports Sci. Coach. 13, 1189–1199. doi: 10.1177/1747954118785503

[ref59] Pomares-Noguera C. Ayala F. Robles-Palazón F. J. Alomoto-Burneo J. F. López-Valenciano A. Elvira J. L. L.. (2018). Training effects of the FIFA 11+ kids on physical performance in youth football players: a randomized control trial. Front. Pediatr. 6:40. doi: 10.3389/fped.2018.00040, PMID: 29556489 PMC5844920

[ref60] Ramirez-Campillo R. Garcia-Hermoso A. Moran J. Chaabene H. Negra Y. Scanlan A. T. (2020). The effects of plyometric jump training on physical fitness attributes in basketball players: A meta-analysis. J. Sport Health Sci. doi: 10.1016/j.jshs.2020.12.005 [Epub ahead of print], PMID: 33359798 PMC9729929

[ref61] Read P. J. Oliver J. L. De Ste Croix M. B. A. Myer G. D. Lloyd R. S. (2016). The scientific foundations and associated injury risks of early soccer specialisation. J. Sports Sci. 34, 2295–2302. doi: 10.1080/02640414.2016.1173221, PMID: 27120711 PMC5500865

[ref62] Read P. J. Oliver J. L. De Ste Croix M. B. A. Myer G. D. Lloyd R. S. (2018). An audit of injuries in six English professional soccer academies. J. Sports Sci. 36, 1542–1548. doi: 10.1080/02640414.2017.1402535, PMID: 29125037

[ref63] Ribeiro J. Davids K. Silva P. Coutinho P. Barreira D. Garganta J.. (2021). Talent development in sport requires athlete enrichment: contemporary insights from a nonlinear pedagogy and the athletic skills model. Sports Med. 51, 1115–1122. doi: 10.1007/s40279-021-01437-6, PMID: 33675517

[ref64] Rudd J. R. Pesce C. Strafford B. W. Davids K. (2020). Physical literacy - a journey of individual enrichment: an ecological dynamics rationale for enhancing performance and physical activity in all. Front. Psychol. 11:1904. doi: 10.3389/fpsyg.2020.01904, PMID: 32849114 PMC7399225

[ref65] Santos E. J. A. M. Janeira M. A. A. S. (2008). Effects of complex training on explosive strength in adolescent male basketball players. J. Strength Cond. Res. 22, 903–909. doi: 10.1519/JSC.0b013e31816a59f2, PMID: 18438223

[ref66] Savelsbergh G. J. P. Wormhoudt R. (2018). Creating adaptive athletes: the athletic skills model for enhancing physical literacy as a foundation for expertise. Mov. Sport Sci. Sci. Mot. 102, 31–38. doi: 10.1051/sm/2019004

[ref67] Smith W. (2016). Fundamental movement skills and fundamental games skills are complementary pairs and should be taught in complementary ways at all stages of skill development. Sport Educ. Soc. 21, 431–442. doi: 10.1080/13573322.2014.927757

[ref68] Strafford B. W. Davids K. North J. S. Stone J. A. (2020). Designing parkour-style training environments for athlete development: insights from experienced parkour traceurs. Qual. Res. Sport Exerc. Health 13, 390–406. doi: 10.1080/2159676X.2020.1720275

[ref69] Strafford B. W. Davids K. North J. S. Stone J. A. (2021). Effects of functional movement skills on parkour speed-run performance. Eur. J. Sport Sci., 1–9. doi: 10.1080/17461391.2021.1891295 [Epub ahead of print], PMID: 33583349

[ref70] Strafford B. W. van der Steen P. Davids K. Stone J. A. (2018). Parkour as a donor sport for athletic development in youth team sports: insights through an ecological dynamics lens. Sports Med. Open 4:21. doi: 10.1186/s40798-018-0132-5, PMID: 29797285 PMC5968018

[ref71] Taylor J. B. Ford K. R. Nguyen A.-D. Terry L. N. Hegedus E. J. (2015). Prevention of lower extremity injuries in basketball: a systematic review and meta-analysis. Sports Health 7, 392–398. doi: 10.1177/1941738115593441, PMID: 26502412 PMC4547118

[ref72] Travassos B. Araújo D. Davids K. (2018). Is futsal a donor sport for football?: exploiting complementarity for early diversification in talent development. Sci. Med. Football 2, 66–70. doi: 10.1080/24733938.2017.1390322

[ref73] Wang X. M. Bingham G. P. (2019). Change in effectivity yields recalibration of affordance geometry to preserve functional dynamics. Exp. Brain Res. 237, 817–827. doi: 10.1007/s00221-018-05467-x, PMID: 30610264

[ref74] Williams M. D. Hammond A. M. Moran J. (2021). Youth basketball coaches’ perceptions and implementation of fundamental movement skills training: toward a realist evaluation. J. Teach. Phys. Educ., 1–8. doi: 10.1123/jtpe.2020-0306, [Epub ahead of print]

[ref75] Witfeld J. Gerling I. E. Pach A. Witfeld J. (2011). Parkour and Freerunning: Discover Your Possibilities. Aachen, Germany: Meyer & Meyer.

[ref76] Witt J. K. Riley M. A. (2014). Discovering your inner Gibson: reconciling action-specific and ecological approaches to perception–action. Psychon. Bull. Rev. 21, 1353–1370. doi: 10.3758/s13423-014-0623-4, PMID: 24683098

[ref77] Woods C. T. McKeown I. Keogh J. Robertson S. (2017). The association between fundamental athletic movements and physical fitness in elite junior Australian footballers. J. Sports Sci. 36, 445–450. doi: 10.1080/02640414.2017.1313996, PMID: 28406356

[ref78] Woods C. T. McKeown I. Rothwell M. Araújo D. Robertson S. Davids K. (2020). Sport practitioners as sport ecology designers: how ecological dynamics has progressively changed perceptions of skill “acquisition” in the sporting habitat. Front. Psychol. 11:654. doi: 10.3389/fpsyg.2020.00654, PMID: 32390904 PMC7194200

[ref79] Wormhoudt R. Savelsbergh G. J. P. Teunissen J. W. Davids K. (2018). The Athletic Skills Model: Optimizing Talent Development Through Movement Education. Oxfordshire, England: Routledge.

[ref80] Young W. B. (2006). Transfer of strength and power training to sports performance. Int. J. Sports Physiol. Perform. 1, 74–83. doi: 10.1123/ijspp.1.2.74, PMID: 19114741

[ref81] Young W. B. Dawson B. Henry G. J. (2015). Agility and change-of-direction speed are independent skills: implications for training for agility in invasion sports. Int. J. Sports Sci. Coach. 10, 159–169. doi: 10.1260/1747-9541.10.1.159

[ref82] Zwolski C. Quatman-Yates C. Paterno M. V. (2017). Resistance training in youth: laying the foundation for injury prevention and physical literacy. Sports Health 9, 436–443. doi: 10.1177/1941738117704153, PMID: 28447880 PMC5582694

